# Factors Associated with Burnout in Healthcare Professionals

**DOI:** 10.3390/ijerph192214701

**Published:** 2022-11-09

**Authors:** Sabinne Marie Taranu, Adina Carmen Ilie, Ana-Maria Turcu, Ramona Stefaniu, Ioana Alexandra Sandu, Anca Iuliana Pislaru, Ioana Dana Alexa, Calina Anda Sandu, Tudor-Stefan Rotaru, Teodora Alexa-Stratulat

**Affiliations:** 1 Department of Medical Specialties II, Faculty of Medicine, Grigore T. Popa University of Medicine and Pharmacy, 700115 Iasi, Romania; 2 Department of Medical Specialties III, Faculty of Medicine, Grigore T. Popa University of Medicine and Pharmacy, 700115 Iasi, Romania; 3 Department of Medical Oncology–Radiotherapy, Faculty of Medicine, Grigore T. Popa University of Medicine and Pharmacy, 700115 Iasi, Romania

**Keywords:** burnout, burnout factors, healthcare professionals

## Abstract

Burnout in healthcare professionals remains an ongoing concern. There are a number of variables associated with reactivity to stress in healthcare staff. This study wants to identify risk factors which predispose healthcare professionals to burnout. Material and Methods: The cross-sectional study included a group of 200 subjects, medical staff and auxiliary staff from the national health units, who gave their free consent to answer the questions regarding the level of perceived stress at work. The screening tool used was disseminated through the Google Forms platform, maintaining the anonymity of the participants. Results: Resident doctors (42%) responded predominantly, reporting the highest level of burnout, with nurses (26.5%) being the least affected (χ^2^ = 36.73, *p* < 0.01). Less work experience is correlated with increased burnout (rho = 0.29, *p* < 0.01). Reactivity to stress was highly associated with workplace, with ambulance staff being the most vulnerable (χ^2^ = 6.58, *p* < 0.05). Participants’ relationship status significantly influenced the burnout rate, the unmarried, with or without a partner, being more affected (χ^2^ = 16.14, *p* < 0.01). There are no significant differences between male and female gender, regarding the average level of burnout (U = 1.47; *p* > 0.05), nor between living in a house or apartment (U = 4.66; *p* > 0.05). Positive associations were identified between the level of burnout and variables such as: management pressure, administrative work, routine, regretting decisions regarding patients, harassment at work and sacrifice of personal time. Conclusions: The results of this study identify age, profession, workplace seniority and relationship status as factors associated with burnout in medical personnel.

## 1. Introduction

Burnout syndrome has always been a challenge for science. Despite ongoing debates, this topic continues to attract the attention of researchers. There are significant effects of burnout to mental and physical heaths, and there are numerous modifiable predisposing factors for burnout that can be easily identified and prevented.

Defining burnout was a long-lasting process, and was initiated from the need to consolidate clear benchmarks for an ambiguous pathology, amenable to difficult differential diagnosis. In 2007, Bakker and Demerouti viewed burnout as a dual model, including increased demands and decreased resources in explaining its occurrence [1,2]. In 2016, Maslach and Leiter achieved a concise delineation, defining burnout as a delayed response reaction to interpersonal stressors occurring at work [1,3,4]. The theory integrates the three subsidiary dimensions of stress reactivity, namely burnout, cynicism and loss of workplace effectiveness. In 2017, the theory is substantiated by Hu et al. They identify a determinism between increased resources, commitment and avoidance of burnout [1,5].

In the health system, stress reactivity has been predominantly correlated with a series of variables related to the work environment, and variables independent of it. When considering variables related to work, we must review the multiple and complex interactions of this professional entity. Concern about the factors associated with burnout in healthcare workers is frequently found in the current literature. A meta-analysis reported many work-related factors for burnout: work-life imbalance, concerns about patients’ conditions, medical specialty, professional degree and training. Financial problems, demographic aspects and associated comorbidities were recorded as stressors independent of the workplace [6].

Exhaustion, depression, anxiety, decreased professional satisfaction, excessive use of certain pharmacological substances, stigmatization and young age have been shown to be important predictors of the burnout syndrome, which seems to have a fulminant evolution, with a series of fatal short-term consequences, among which are suicidal tendencies [7,8,9]. Data are showing an increase in stress levels in young medical staff with more than one depressive episode per week [10]. Other data have speculated on the importance of the intelligence phenotype in the prevention or development of burnout in medical personnel. Thus, emotional intelligence appears to play an important role in mitigating stress reactivity [11].

Female genetic sex in healthcare professionals can be predisposed to burnout. Different data show a higher disposition to physical exhaustion and depersonalization of the medical act in female nurses working in the ambulance compared to male nurses or doctors working in the same conditions [12,13]. In addition, another study presents the high chances of developing burnout syndrome in female resident doctors, who work more than 80 h a week, regardless of their specialization [14].

Workplace location, urban vs. rural areas, is also significant in the development of burnout. The literature also shows a predisposition to burnout for urban workers, thus having a higher tendency for quitting. A number of factors are cited to interfere with the stress reactivity of medical personnel working in large cities, such as: low incomes in relation to high prices, increased traffic and increased competition in the field of activity [15].

A 2020 Chinese meta-analysis classified medical staff stressors into systemic (work environment, health care system, medical culture), personal (unhealthy perfectionism, exaggerated altruism, self-reproach), and interpersonal (degree of empathy, marginalization, ethical order distress). Empathy, altruism and attachment in a professional environment, driven to maximum levels, determine emotional and cognitive damage, with more negative effects in the long term [16]. Much new data focus on cognitive emotion regulation strategies. Among these, the phenomenon of empathizing plays an important role. The phenomenon of empathizing goes both ways: on one hand, it increases the quality of the professional work through compassion; on the other hand, it can decrease the satisfaction at work by fueling feelings of helplessness and guilt [17]. In the medical field, unhealthy empathy culminates in emotional exhaustion and depersonalization of the relationship with the patient, also negatively influencing safety, health and individual resilience [17,18].

Burnout affects all healthcare workers. Data about the most affected medical personnel (doctors, nurses, orderly, technical auxiliary staff such as registrar, secretary, electrician, etc.) differ from study to study. A previous study shows a higher tendency to physical exhaustion of nurses working in the emergency department, compared to doctors, with no differences between the two categories regarding the loss of devotion to the workplace and the depersonalization of the medical act [12]. According to other data, it seems that subordinate medical personnel, working with limited resources and in poor conditions, have a low quality of life and dehumanize themselves, with development of cynicism, loss of commitment and the onset of burnout [19,20]. There are data that strictly delimit an increased predisposition to burnout in nurses and doctors, compared to auxiliary staff, explained also accounting for interaction and empathy with patients and their families [21,22,23,24]. Other results show a high prevalence of loss of professionalism and physical exhaustion in nurses and orderly, by constantly confronting the deficiencies of the administrative and technological system [25]. There are studies that also focus on the level of burnout among medical staff in hospitals, drawing attention to some precipitating factors such as: the way the hospital is administered, permanent supervision by superiors, overtime working hours and poor relations with the professional group [26,27,28,29,30,31]. Therefore, it seems that, for medical staff who work in a united team and believe in the effectiveness of teamwork, regardless of increased workloads or even if they work in an ambulance, the burnout rate is lower [32].

Low work domain experience is significantly correlated with increased stress reactivity. Information from the literature supports our findings, showing the role of tenure and level of professional training in delaying the onset of burnout [33]. Along with increased experience in the field and older age, seniority is correlated with lower burnout rates, an aspect also indirectly supported by our results [34,35,36].

Burnout has important repercussions on the quality of the medical act, healthcare costs and individual health. The medical staff burnout syndrome has a number of negative consequences for patients as well, such as: increasing the risk of medical errors, jeopardizing the relationship with the patient and, consequently, compromising the quality of the medical act. Interventions to prevent the onset of burnout syndrome, with its screening from the early stages remaining the fundamental objectives in the therapy plan, targeting both general strategies and principles of individualized approaches [3,37].

The correct recognition of potential stress reactivity trigger factors, related or not to the activity environment, can be considered a significant step to prevent burnout. Thus, in the early stages of its debut, the recognition of the predisposing factors represents a method of risk forecasting, a form of prophylaxis, and in the case of severe burnout, a way of reducing mental tension [3].

Despite the fact that all the relevant literature legitimizes the burnout syndrome with its maintenance factors, the recourse to these data needs strengthening. In the medical system, the constant reshaping of stress conditions requires a permanent update, and the aspects associated with burnout always remain open to research [38,39].

Our study’s objectives are to identify the burnout level in the healthcare professionals and to determine the factors associated with burnout in the healthcare professionals. Once these factors are identified, it is easier to prevent or treat in order to decrease burnout in the healthcare professionals. There are few studies in our country/region for burnout in healthcare professionals. Our study intends to fill the gap and identify the particularities for our population.

## 2. Material and Methods

We present a cross sectional study that started in September 2021 and was continued until March 2022. The study included a group of 200 subjects, medical staff and auxiliary staff from national health units, who have freely consented to answer the questions regarding the level of perceived stress at work. We developed a questionnaire to determine burnout in medical personnel. We included items from one of the first burnout screening tools developed by Katrina Shields [40], items from the Maslach Burnout Inventory [41] and our own items. Our own items were identified in previous studies as factors for developing burnout. In addition, we included pandemic-related factors, but the results are in process.

The screening tool used was disseminated online, through the Google Forms platform, while maintaining the anonymity of the participants. The questionnaire was distributed online for two reasons: the study was conducted in COVID-19 pandemic, and we wanted to have answers from a greater geographical region. Every participant was asked to provide an email, which remained confidential. All participants completed the informed consent. The questionnaires were distributed after obtaining the approval of the Ethics Commission.

Data were collected by our study’s team of physicians. All the answers were collected in a single file and analyzed using SPSS 18.0 software. Internal consistency was calculated for our burnout questionnaire. The Alpha Crombach coefficient was excellent at 97. All scale values were summed, and normality of distribution was tested. The distribution of total burnout scores varied significantly, by reference to the normal curve, as indicated by the Kolmogorov–Smirnov normality test (Z = 1.59, *p* < 0.05). Therefore, no parametric tests were used, only non-parametric tests.

ANOVA test, F test (ANOVA), multivariate analysis, ROC curve, Student’s *t*-test, Kruskal–Wallis, Mann–Whitney and Spearman correlations were applied in quantitative data interpretation. Statistical significance was defined at the 95% confidence interval (*p* < 0.05).

## 3. Results

**Figure 1:**
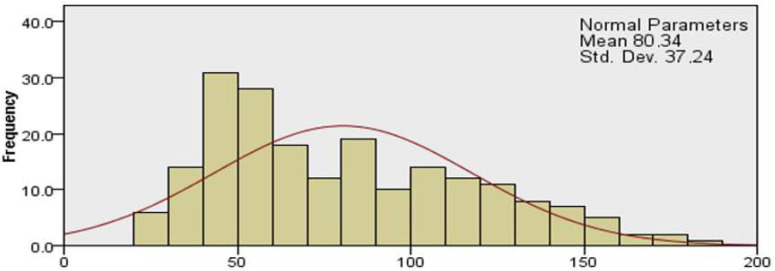
Total burnout score for healthcare professionals.

The study group included 200 subjects, and their demographics are represented in Table 1. Total burn out score was of 80.34 ± 37.24. (Figure 1.)

### 3.1. Age

Significant negative Spearman correlations were identified between the level of burnout and the age of the research participant (r = −0.27, *p* < 0.01) (Table 2).

### 3.2. Gender

**Figure 2:**
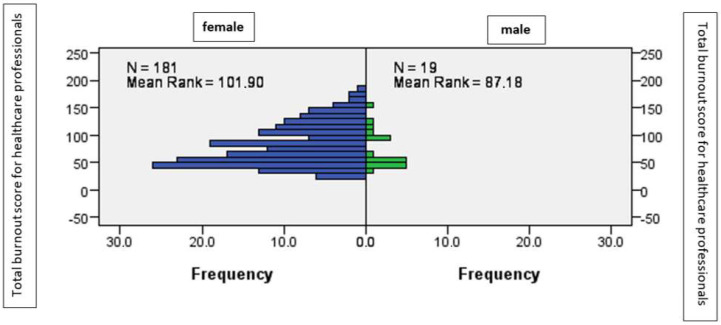
The average level of burnout according to gender.

There were no significant differences between the male and female genetic sex, regarding the average level of burnout, as shown by the Mann–Whitney statistic (U = 1.47; *p* > 0.05), (Figure 2).

### 3.3. Workplace Location

**Figure 3:**
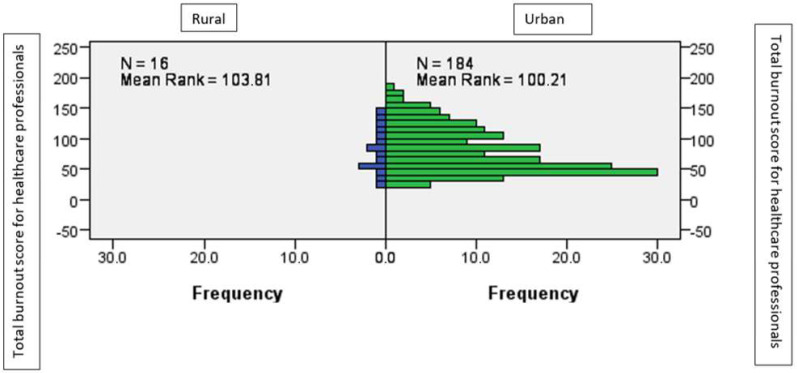
The average level of burnout according to workplace location.

There are no significant differences between those who worked in the urban environment and those who worked in the rural environment, in terms of the average level of burnout, as shown by the Mann–Whitney statistic (U = 1.42; *p* > 0.05) (Figure 3).

### 3.4. Profession

**Figure 4:**
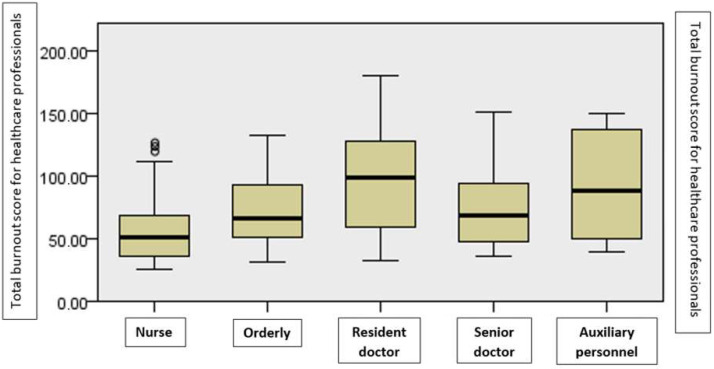
The average level of burnout by profession.

There are significant differences between the various groups determined by the profession, in terms of burnout, as shown by the non-parametric Kruskal–Wallis test for the difference between the mean rank (χ^2^ = 36.73, *p* < 0.01). Medical assistants reported the lowest level of burnout, while medical residents reported the highest level of burnout (Figure 4).

### 3.5. Work Type

**Figure 5:**
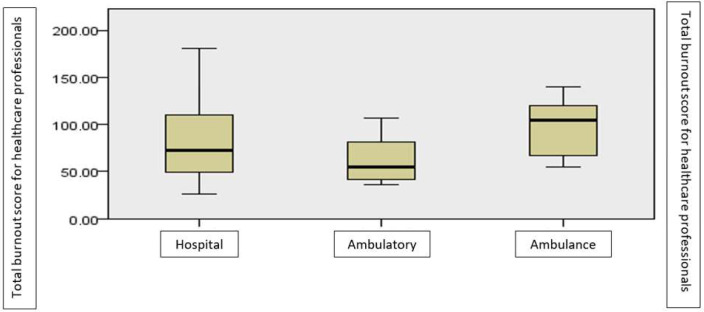
The average level of burnout according to the work type.

There are significant differences between the various groups according to the work type, in terms of burnout, as shown by the non-parametric Kruskal–Wallis test for the difference between the mean rank (χ^2^ = 6.58, *p* < 0.05). The highest level of burnout is reported by those working on ambulance, followed by those in the hospital, then by those in the ambulatory (Figure 5).

### 3.6. Workplace Seniority

There is a significant correlation between the year of starting the activity and the level of burnout (r = 0.29, *p* < 0.01), which shows a higher level of burnout in younger medical professionals (Figure 4, Table 2).

### 3.7. Relationship Status

**Figure 6:**
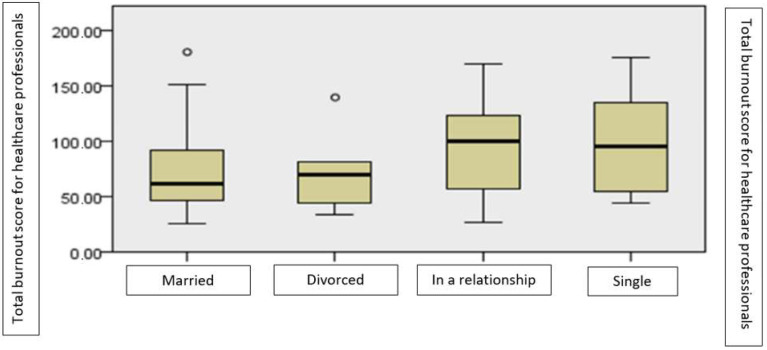
The average level of burnout according to participants’ relationship status.

There are significant differences between the various groups determined by participants’ relationship status, in terms of burnout, as shown by the non-parametric Kruskal–Wallis test for the difference between the mean rank (χ^2^ = 16.14, *p* < 0.01). The highest level of burnout is reported by those who are single, with or without a partner (Figure 6).

### 3.8. Persons in One’s Care

Significant negative Spearman correlations were identified between the level of burnout and the number of children in one’s care (r = −0.29, *p* < 0.01).

### 3.9. Other Factors

For all variables with at least a three-step response scale in the burnout-related variables section, Spearman correlations were run with the total burnout score. The answers to the following questions correlated significantly positively with the burnout score of the medical staff (Table 3).

Based on Spearman correlations (rho+ (r)), directly proportional and positively significant associations (*p* < 0.01) were identified between the level of burnout and factors associated with burnout in medical personnel, such as: pressure from management (r = 488), administrative work (r = 495), conflictual relations with colleagues (r = 309), fear of fatal mistakes for the patient (r = 328), pressure of responsibility towards patients (r = 326), excessive bureaucracy (r = 443), routine at work (r = 446), lack of time to perform tasks (r = 375), regretting some decisions made regarding patients (r = 311), harassment at work (r = 488), loss of professionalism (r = 444), tendency to compromise (r = 546) and personal time allocated to patients (r = 343).

## 4. Discussion

In our study, factors associated with burnout were age, profession, workplace seniority, relationship status and the presence of persons in care. We found that, the younger the age of the participant, the higher risk for burnout. This has a connection with the profession factor in our study because resident doctors are more predisposed to burnout. In addition, we found that the participants with less work experience are more predisposed to burnout. Another finding is that married respondents were less predisposed to burnout, and those with children were also less predisposed.

Age is a factor associated with burnout in our study. This finding is also cited by previous studies [10]. There are multiple explanations: lack of experience, higher empathy and longer work hours. At the same time, the increased number of nightshifts and working overtime are factors often cited in the literature as having an important role in generating burnout in resident doctors, regardless of the specialization they choose [14]. In addition, a particular aspect from our country is the tendency for resident doctors to deflect to other countries. This fact is causing a stalemate, with the workload increasing for the residents who remain [42,43].

Resident doctors presented the highest level of burnout in our study. One explanation is residents’ lack of work experience, also an important factor for burnout. A previous study that included psychiatry physicians and residents showed that the resident doctors have a high prevalence of burnout, depersonalization, and a decrease in effectiveness at work. The economic factor does not statistically influence the results [39]. On the other hand, the fact that the resident doctors were the higher respondents in our study may lead to a higher statistical power.

Contrary to our study where nurses were the least predisposed to burnout, literature is evocative in signaling the predisposition to burnout in the medical personnel, especially in nurses [44,45,46]. A study that included nurses demonstrated a positive correlation between job competence, salary and job satisfaction. Workplace efficiency has been negatively influenced by abuse and burnout [46,47].

According to other data, clinicians and nurses who were exposed to physical and non-physical violence in the workplace have associated increased rates of depression and anxiety. Nurses were more affected than doctors [47].

Other results show a statistically significant correlation between the phenomenon of rumination, more frequently recorded in nurses, and the depersonalization of the medical act, as a predictor of the burnout syndrome [18,48].

A study of nurses and geriatric nurses also found significant associations between stress reactivity sub-entities. Thus, emotional exhaustion was most frequently associated with the development of severe burnout, and lack of job satisfaction was correlated with the early stage of burnout [18].

Our results identify a directly proportional correlation between increased burnout and decreased work experience. A Spanish multicenter analysis confirms our data, highlighting that, in a group of nurses, the low number of years of experience was more frequently related to the tendency to develop burnout [48]. Another study, conducted at the level of geriatric staff, contradicts our results, citing long experience in interacting with elderly patients in positive correlation with the depersonalization of the medical act and with the decrease in professionalism [18]. Consistent with our data, low work domain experience is significantly correlated with increased stress reactivity. Information from the literature supports our findings, showing the role of tenure and level of professional training in delaying the onset of burnout [33]. Along with increased experience in the field and older age, seniority is correlated with lower burnout rates, an aspect also indirectly supported by our results [34,35,36].

In our study, unmarried participants with or without a partner had the highest level of burnout. In recent literature, there are more specific data showing a positive correlation between female gender, single relationship status and stress reactivity; therefore, these factors were associated with burnout [49]. However, not all studies confirm relationship status, and more specifically not being married, as a determinant for burnout [50,51]. There are also data showing higher levels of stress in married or widowed physicians than in single or divorced physicians, stress that contributes to burnout [52]. Furthermore, studies do not show a connection between relationship status and the onset of burnout [53,54].

Our data suggest a higher level of burnout in participants with fewer persons in their care. We explain this aspect considering the resilience level that family can induce. We believe that caregivers find a balance between attention to the profession and attention to the family.

Work type (ambulance, hospital, ambulatory) is also a factor associated with burnout, but not with the same statistical significance. According to the results of our study, those who work in an ambulance showed an increased reactivity to stress, compared to those who work in hospitals or in an outpatient environment. Most recent studies confirm this aspect. A French study carried out on different medical specialties objectified that burnout predominantly affected doctors in the emergency medicine specialization. Among residents, a higher rate of medical depersonalization was reported. The increased number of nightshifts per month was associated with a decrease in workplace efficiency. A low rate of burnout, but an increased rate of depersonalization of the medical act, was observed among anesthesiologists [55]. Another study, conducted between 2011 and 2014, observed an increase in the burnout rate in emergency medicine physicians over time [56]. Other recent data report an increased tendency to develop burnout in a cohort of emergency physicians, with alcoholism identified as a long-term consequence. In addition, in them, the role of the increased number of working hours in cognitive impairment was highlighted, with the appearance over time of obesity, anxiety and decreased performance at work [37]. Some data show that ambulance medical personnel develop burnout in the context of higher demands and more pronounced physical overwork, registering a higher tendency towards desertion in this category [57].

Other studied factors associated with burnout in previous studies did not reach statistical significance in ours. These factors are: genetic sex and workplace location (urban area, rural area).

The majority of our study group was represented by the female sex (90.5%). We translate the fact that there were no significant differences between men and women, considering the average level of burnout, but further studies are needed with more balanced genetic sex distribution. Moreover, the literature incriminates the female gender in the predisposition to burnout. In this sense, a recent meta-analysis showed that the female gender, young age and concern related to the evolution of patients are the highly predisposing factors for burnout in medical personnel [7]. In addition, other data indicated that suicidal ideation and suicide attempt were recorded in those in the medical field, with advanced age and female sex [16,38].

In addition, our data show negative associations between the level of burnout and the age of the participant, the number of rooms in the house, the number of people with whom the person lives and the number of children in care. Previous studies are inconsequently signaling both similar results, by identifying young age as a factor associated with burnout, and different ones, by highlighting burnout symptomatology in older people [38,56].

Studies speculate differently on the role of medical specialization in precipitating the burnout syndrome [58]. There is also much data that expose the prevalence of emotional exhaustion and loss of professionalism in surgical specialties. However, each specialization can generate specific factors for maintaining mental tension [59].

Our data predict a tendency for those who live in apartments to develop more significant burnout, the statistic being at the limit of the threshold of significance. The results are similar to those of other studies that certify a significantly higher burnout rate in those who live in apartments [53].

Among the variables related to medical staff burnout, administrative work and excessive bureaucracy were significantly positively associated with stress reactivity. Among the risk factors for burnout is also the technologization of the administrative field, with frequent impasses occurring because of medical IT program issues. Comparably, a recent study, enrolling participants from the specialties of geriatrics and family medicine, describes this aspect in detail [60].

We also identify a positive association between sacrificing personal time for the benefit of the patient and the predisposition to burnout. The results of a meta-analysis published in 2020 mostly report this association [8].

Our study has some limitations: (1) number of participants and the inequal proportions among healthcare workers, as nurses, auxiliary staff and male participants responded in very small numbers. We consider this limitation in the context of choosing an online and anonymous survey method, with random participation and also, in our country, at the national level, each health unit depends on a smaller number of representatives of the auxiliary technical staff (registrar, secretary, electrician, etc.) and more representatives of the healthcare workers. (2) We cannot completely eliminate de risk for duplicate participants. We asked for all participants mail, but considering the online questionnaire we do not have a certainty for nonduplicate participants. (3) Our study was conducted in COVID-19 pandemic curfew period. The results of the study may interfere with the restrictions and changes generated by the COVID-19 pandemic and its impact on the medical system. The results regarding the role of the pandemic in precipitating the burnout syndrome, are in process and will be the subject of a future article.

## 5. Conclusions

Age, profession, workplace seniority and relationship status are factors associated with burnout in our population. The importance of identifying burnout factors in medical personnel resides in preventing the onset of the burnout syndrome or reducing it [2,3,4].

Due to permanent changes in the healthcare field, the conditions for mental tension and burnout change simultaneously with the reactivity to stress [60]. The technologization of the medical system, the constant adjustment of intra-hospital circuits and the frequent reconfiguration of job requirements in relation to staff shortage are a few of the new factors associated with burnout identified in our study.

It is very important to identify the variables not influenced by the work environment such as: female gender, young age, relationship status, living in an apartment and those specific to the workplace, such as medical profession or work place, because all play an essential role in determining the risk for burnout.

Admitting the variables related to increased stress reactivity constitutes an increased possibility of stress level predictability. Thus, we must identify in the healthcare professionals the burnout factors such as tendency to compromise, loss of professionalism and pressure of responsibility towards the patient. At the same time, the recognition of these factors must be accompanied by adapted measures to prevent the burnout in the healthcare professionals, including continuing education regarding burnout and interventions for increasing individual resilience.

## Figures and Tables

**Table 1:** General data and demographic aspects of the study group.

	Total Number (Percentage)	Mean ± Standard Deviation
Age		36.09 ± 9.364
Sex		
Women	181 (90.5%)	
Men	19 (9.5%)	
Workplace location		
Rural	16 (8%)	
Urban	184 (92%)	
Profession		
Senior doctor	53 (26.5%)	
Resident doctor	84 (42%)	
Nurse	53 (26.5%)	
Orderly	6 (3%)	
Auxiliary personnel	4 (2%)	
Work type		
Hospital	176 (88%)	
Ambulatory	16 (8%)	
Ambulance	8 (4%)	
Workplace seniority		2011.95 ± 10.112
Relationship status		1.95 ± 1.159
Married	113 (56.5%)	
Divorced	10 (5%)	
In a relationship	51 (25.5%)	
Single	26 (13%)	
Persons in care		
Children		0.62 ± 0.8055
Seniors		0.245 ± 0.5888
Total burnout score		
Medical staff		80.34 ± 37.24

Correlations between the level of burn-out and the studied parameters.

**Table 2:** Correlations between the level of burn-out and the studied parameters.

	*p*	r (rho)
Age	<0.001	−0.266
Genetic sex	0.292	1.47
Workplace location	0.811	1.42
Profession	<0.001	36.734
Work type	0.037	6.58
Workplace seniority	<0.001	0.29
Relationship status	<0.001	16.14
Persons in care (number)	0.016	−0.171
Persons in care	<0.001	−0.29
Children	<0.001	−0.293
Seniors	0.546	−0.043

**Table 3:** Correlations between factors associated with burnout and the total burnout score.

	*p*	r
Total burnout level score for the medical staff	0.01	1000
I feel a lot of pressure from the hospital management.	0.01	488
It bothers me that I have to deal with the administrative side as well, not just with patients.	0.01	495
At work, I encounter conflict situations with my colleagues.	0.01	309
I am constantly afraid of making a mistake that could cost a patient’s life.	0.01	328
I feel the pressure of the responsibility I have towards patients all the time.	0.01	326
I deal more with medical documents than with patients.	0.01	443
I face a routine in the activities carried out at work.	0.01	446
I always feel that I have to do many things in a short time.	0.01	375
I feel regret for the decisions I made in the cases of certain patients.	0.01	311
I felt harassed at work.	0.01	488
I feel overwhelmed in certain cases and cannot react professionally.	0.01	444
I feel that I am not completely correct in the professional environment, having to make certain compromises.	0.01	546
In my free time, I always think about patients’ cases.	0.01	343
